# Longitudinal Evaluation of Plasma Cytokine Levels in Patients with Invasive Candidiasis

**DOI:** 10.3390/jof7020101

**Published:** 2021-02-01

**Authors:** Stefanie Wunsch, Christoph Zurl, Heimo Strohmaier, Andreas Meinitzer, Jasmin Rabensteiner, Wilfried Posch, Cornelia Lass-Flörl, Oliver Cornely, Gudrun Pregartner, Elisabeth König, Gebhard Feierl, Martin Hoenigl, Juergen Prattes, Ines Zollner-Schwetz, Thomas Valentin, Robert Krause

**Affiliations:** 1 Section of Infectious Diseases and Tropical Medicine, Department of Internal Medicine, Medical University of Graz, 8036 Graz, Austria; christoph.zurl@medunigraz.at (C.Z.); elisabeth.ullrich@medunigraz.at (E.K.); mhoenigl@health.ucsd.edu (M.H.); juergen.prattes@medunigraz.at (J.P.); ines.schwetz@medunigraz.at (I.Z.-S.); thomas.valentin@klinikum-graz.at (T.V.); 2 BioTechMed-Graz, 8010 Graz, Austria; 3 Department of Paediatrics and Adolescent Medicine, Division of General Paediatrics, Medical University of Graz, 8036 Graz, Austria; 4 Center for Medical Research, Medical University of Graz, 8010 Graz, Austria; heimo.strohmaier@medunigraz.at; 5 Clinical Institute of Medical and Chemical Laboratory Diagnostics, Medical University of Graz, 8036 Graz, Austria; andreas.meinitzer@medunigraz.at (A.M.); jasmin.rabensteiner@medunigraz.at (J.R.); 6 Institute of Hygiene and Medical Microbiology, Medical University of Innsbruck, 6020 Innsbruck, Austria; wilfried.posch@i-med.ac.at (W.P.); cornelia.lass-floerl@i-med.ac.at (C.L.-F.); 7 Excellence Center for Medical Mycology (ECMM), Department I of Internal Medicine, Faculty of Medicine and University Hospital Cologne, University of Cologne, 50937 Cologne, Germany; oliver.cornely@ctuc.de; 8 Chair Translational Research, Cologne Excellence Cluster on Cellular Stress Responses in Aging-Associated Diseases (CECAD), Faculty of Medicine and University Hospital Cologne, University of Cologne, 50931 Cologne, Germany; 9 Clinical Trials Centre Cologne (ZKS Köln), Faculty of Medicine and University Hospital Cologne, University of Cologne, 50935 Cologne, Germany; 10 Institute for Medical Informatics, Statistics and Documentation, Medical University of Graz, 8036 Graz, Austria; gudrun.pregartner@medunigraz.at; 11 Diagnostic & Research Institute of Hygiene, Microbiology and Environmental Medicine, Medical University of Graz, 8010 Graz, Austria; gebhard.feierl@medunigraz.at; 12 Division of Infectious Diseases and Global Public Health, Department of Medicine, University of California San Diego, San Diego, CA 92093, USA

**Keywords:** interleukin 17A, interleukins, cytokines, biomarker, *Candida*, invasive candidiasis, candidemia, tryptophan-kynurenine metabolism

## Abstract

Interleukin (IL) 17A plays a decisive role in anti-*Candida* host defense. Previous data demonstrated significantly increased IL-17A values in candidemic patients. We evaluated levels and time courses of IL-17A, and other cytokines suggested to be involved in *Candida*-specific immunity (IL-6, IL-8, IL-10, IL-17F, IL-22, IL-23, interferon-γ, tumor necrosis factor-α, Pentraxin-related protein 3, transforming growth factor-β) in patients with invasive candidiasis (IC) compared to bacteremic patients (*Staphylococcus aureus*, *Escherichia coli*) and healthy controls (from previous 4 days up to day 14 relative to the index culture (−4; 14)). IL-17A levels were significantly elevated in all groups compared to healthy controls. In IC, the highest IL-17A values were measured around the date of index sampling (−1; 2), compared to significantly lower levels prior and after sampling the index culture. Candidemic patients showed significantly higher IL-17A values compared to IC other than candidemia at time interval (−1; 2) and (3; 7). No significant differences in IL-17A levels could be observed for IC compared to bacteremic patients. Candidemic patients had higher IL-8, IL-10, IL-22, IFN-γ, PTX3 and TNF-α values compared to non-candidemic. Based on the limited discriminating competence between candidemia and bacteremia, IL-17A has to be considered a biomarker for blood stream infection rather than invasive *Candida* infection.

## 1. Introduction

Globally, *Candida* species (spp.) rank as the fourth most common cause of nosocomial bloodstream infections [1,2,3,4]. Invasive candidiasis (IC) is associated with substantive mortality rates ranging from 5% to more than 70% [2,5,6,7,8]. Since invasive *Candida* infections primarily occur in patients with serious underlying diseases, it is often difficult to differentiate between attributable mortality of candidemia and mortality owing to comorbidities [5]. Timely diagnosis and subsequent initiation of antifungal therapy are crucial for survival in patients with IC [5,6,9,10,11,12,13,14], since a delay of one to two days in initiation of adequate antifungal therapy results in doubled mortality rates [1]. However, the clinical diagnosis of IC is complicated by the fact that no signs or symptoms are specific for invasive *Candida* infection [1,11,15]. Furthermore, no single test or decision rule is able to precisely distinguish between contamination, commensalism, colonization or infection [16].

Currently available diagnostic tests include well-known diagnostic methods, such as culture, histopathology, microscopy, antigen detection and polymerase chain reaction (PCR) [1,2,12]. Blood cultures are considered the diagnostic gold standard but have a long turn-around time and limited sensitivity missing approximately 50% of IC cases [2,6,15,17,18,19]. The strength of non-culture-based methods like 1,3-beta-D Glucan testing (BDG) is its high negative predictive values, whereas positive predictive values are low [19]. Up to date, no specific rapid diagnostic approach is available for anticipation or reliable and timely diagnosis of IC. Due to the lack of reliable diagnostic tools, antifungal therapy is often initiated in patients at risk for invasive *Candida* infection after *Candida* spp. have been isolated from various non-sterile body sites [10,11,14,20]. Consequences of this approach include overtreatment of patients, increased antifungal selection pressure with consecutive emergence of antifungal resistance, potential adverse effects, and increased costs [2,11,14].

Interleukin 17A (IL-17A), the major cytokine secreted by T helper type 17 cells, as well as kynurenine, showed time-dependent elevations in candidemic patients compared to non-candidemic patients with significantly higher values in the early course of candidemia compared to later stages [21]. The high discriminating competence of IL-17A values between *Candida*-infected and non-infected patients suggested a potential of IL-17A as a biomarker for timely recognition of IC. In an observational, prospective study, IL-17A levels were shown to be significantly increased in three patients with septic shock due to candidemia (primarily abdominal focus) compared to non-candidemic septic patients with or without *Candida* colonization, supporting the usefulness of IL-17A values for the diagnosis of invasive *Candida* infections [22].

The objective of the present study was to evaluate IL-17A plasma levels and other cytokines as potential biomarkers for early anticipation of IC. Since previous research suggested a time-dependent course of IL-17A levels in patients with IC, which has not been investigated further so far, we performed serial measurements of IL-17A, tryptophan, kynurenine and other cytokines (IL-6, IL-8, IL-10, IL-17F, IL-22, IL-23 (p19), IFN-γ, TNF-α, PTX3, TGF-β), and compared them to bacteremic patients, as well as healthy controls.

## 2. Materials and Methods

This prospective multicenter study was conducted at the Medical University of Graz, Austria (center 1), the Medical University of Innsbruck, Austria (center 2), and the University Hospital of Cologne, Germany (center 3). Recruitment of study patients was performed between September 2015 and December 2018. The study was approved by the local ethics committee (Graz, protocol number 19-322 ex 07/08) and was performed according to the standards of “Good Scientific Practice”. Written informed consent was obtained from all patients eligible for the study. If patients were not able to give written informed consent at the time of study inclusion (e.g., intubated and mechanically ventilated patients), they were informed and asked for their consent after their arousal.

At center 1, adult patients ≥18 years of age were prospectively screened for study inclusion and were assigned to one of four groups: patients with IC, patients with *Staphylococcus aureus* (*S. aureus*) bacteremia, patients with *Escherichia coli* (*E. coli*) bacteremia, and healthy controls. Center 2 and 3 only included candidemic patients.

Diagnostic criteria for proven and probable IC used in our study are represented in Table A1 (see Appendix A). According to the recently revised and updated European Organisation for Research and Treatment of Cancer/Mycoses Study Group (EORTC/MSG) definitions [23], recovery of *Candida* spp. from blood cultures or from a sterile site combined with clinical or radiological abnormality consistent with an infectious disease process was classified as proven IC. Definite candidemia cases (including only patients with *Candida* positive peripheral +/− central blood cultures) were labeled “true candidemia”. However, some of the study patients with candidemia presented only *Candida* positive central blood cultures, but negative peripheral blood cultures, which potentially represents *Candida* colonization of central venous catheters rather than candidemia. Thus, differential time to positivity for determination of catheter-related bloodstream infection could not be calculated in these patients. Consequently, they could not be reliably classified as “true candidemia” and were classified as IC (other).

Cases that met the criteria for a host factor and a clinical feature, yet lacking mycological evidence, were considered possible IC [23].

*S. aureus* and *E. coli* bacteremia was defined as recovery of these organisms from peripheral blood cultures. The control group comprised healthy individuals without underlying (infectious) diseases undergoing elective plastic surgery.

The sampling day (in the further course) of *Candida* spp. positive blood or sterile site cultures, and *S. aureus* or *E. coli* positive blood cultures (=index cultures) was defined as day 1. All of the obtained data refer to this date, unless otherwise stated. Patients with positive *Candida* spp., *S. aureus,* or *E. coli* follow-up cultures during the same hospital stay were counted as one patient. The date of detection of yeasts on Gram stain or *S. aureus* or *E. coli* with fast identification methods (e.g., Matrix Assisted Laser Desorption Ionization—Time of Flight Mass Spectrometry (MALDI-TOF-MS)) was designated as “date of first record” and corresponded to the date of study inclusion. As soon as *Candida* spp., *S. aureus* or *E. coli* was detected in blood cultures or sterile site specimens, retained blood samples were collected. Retained blood samples describe routinely drawn blood samples, which are processed in our in-house clinical chemical laboratory units, and, after determination of requested laboratory values, were stored at 4 °C for up to 4 days in case additional testing was required.

Blood cultures, specimens from normally sterile sites and BDG testing were obtained as clinically indicated at the discretion of the attending physician. Routinely investigated blood cultures and sterile site specimens processed in the in-house microbiology laboratories were monitored daily for the presence of *Candida* spp., *S. aureus* or *E. coli* positivity. In case of positive blood cultures, Gram staining and fast identification methods were performed (i.e., MALDI-TOF-MS; Bruker Maldi Biotyper^®^, Bruker, Vienna, Austria). Patients with *Candida* spp., *S. aureus* or *E. coli* recovered from blood cultures or from sterile site specimens (designated index cultures) were assigned to the respective study groups as described above. CHROMagar™ *Candida* Medium (Becton Dickinson, Heidelberg, Germany) was used for subculturing of *Candida* spp. All isolated *Candida* spp. were identified by MALDI-TOF-MS. Antifungal susceptibility testing of *Candida* spp. was performed using E-test strips according to the manufacturer’s instructions (AB Biotest, Solna, Sweden).

**Figure A1:**
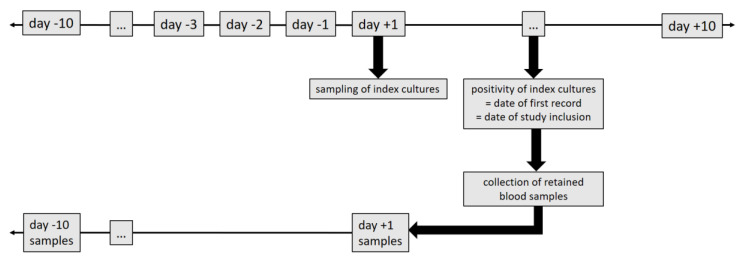
Sampling schedule.

Immediately after study inclusion, all available lithium heparin tubes (Greiner Bio-One™, Kremsmünster, Austria) from routine laboratory investigations (i.e., retained blood samples) of particular study patients were collected from our in-house clinical chemical laboratory unit. Retained blood samples from the sampling day of index cultures were defined as “day 1 samples”. Accordingly, blood samples from the day before were designated as “day 1 samples” and blood samples from the day after were designated as “day 2 samples”, etc. (see Figure A1). Through collection of retained blood samples, preferably before positivity of index cultures, and ideally even before sampling of index cultures, investigation of biomarkers at earlier time points compared to previous studies was intended. Beginning with the date of study inclusion, retained blood samples were collected on a daily basis (if available from routine clinical work-up) until day 14 (Figure A1). A total of 1204 lithium-heparin samples were collected by this procedure. Blood samples were stored at −80 °C until further analysis.

Serum BDG testing was performed using an adapted and automated protocol of the Fungitell^®^ assay as described earlier [24]. According to the manufacturer, a BDG value of ≥80 pg/mL was considered to be positive.

Selection of cytokines was based on previous research from both murine models and clinical studies, showing an increase or decrease of these plasma or serum cytokine levels in patients with *Candida* infections [7,25,26,27,28,29,30,31,32,33,34]. Investigated cytokines were: IL-6, IL-8, IL-10, IL-17A, IL-17F, IL-22, IL-23 (p19 subunit), IFN-γ, TNF-α, PTX3 and TGF-β.

Cytokine values were determined using a customized 11-Plex immunoassay (assay ID: PPX-11-PTX3LAP; ThermoFisher Scientific Inc., Waltham, MA, USA) according to the instructions of the manufacturer. The multiplex suspension bead array immunoassay allows simultaneous measurement of the afore-mentioned cytokines. Standard curves for each analyte were generated using the reference analyte concentration supplied by the manufacturer. The measurement was performed on a calibrated Bio-Plex^®^ 200 system (Bio-Rad, Hercules, CA, USA) in combination with Bio-Plex Manager software, version 6.1 (Bio-Rad). Median fluorescence intensity (MFI) values were considered valid when the bead count reached a minimum of 50 beads per bead set per well. For samples with low bead count, measurements were repeated. Each sample was measured in duplicate and the cytokine concentrations were calculated from the standard curve using 5-Parameter Logistic curve fitting. Measurements were performed at the Center for Medical Research, Medical University of Graz (center 1), as described earlier [35].

Kynurenine and tryptophan concentrations were measured in plasma samples by high-performance liquid chromatography (HPLC) with a simultaneous ultraviolet and fluorometric detection system as described previously [21]. Measurements were performed at one of our in-house clinical chemical laboratory units. Due to limited testing capacities, kynurenine and tryptophan levels were only determined for a randomly selected proportion of patients with IC, as well as *S. aureus* or *E. coli* bacteremia from center 1 (Graz).

The study cohort was descriptively analyzed using medians and ranges (min-max) for continuous parameters and absolute and relative frequencies for categorical parameters. The Chi-squared test was used for the comparison of categorical variables, whereas the Kruskal-Wallis test was used to compare continuous variables between the groups.

Since blood samples were drawn at the discretion of the attending physician, the number of cytokine measurements per patient varied and measurements were not usually available for each day. We therefore investigated differences in interleukins, kynurenine/tryptophan, BDG, leukocytes, C-reactive protein (CRP), and procalcitonin (PCT) between the five study groups regarding specific time intervals (days) defined as follows (day 1 corresponds to the day of index sampling): (−4; −2), (−1; 2), (3; 7), (8; 14). For patients with multiple cytokine measurements from the same day, the first cytokine value of each study day was used for statistical analysis. Regarding healthy controls, only one cytokine value from the date of study inclusion was available, which was repeatedly used for each time interval. Statistical analyses comprised all available data for the defined time intervals. By using linear mixed models (LMM) with random intercept per patient, repeated measurements per patient could be adequately considered. For validation of model assumptions, residual plots were inspected. Due to skewness in the outcome parameters of interest, a log transformation was performed for these analyses. Results are presented as estimates for group differences on the log-scale along with the 95% confidence interval and a corresponding *p*-value. Furthermore, IL-17A values within study group IC (true) were similarly compared between the study intervals.

These analyses were performed for the total study population (main analysis), as well as without immunocompromised patients or patients with hematological malignancies (sensitivity analysis). The statistical analyses were performed using SPSS, version 26 (SPSS, Chicago, IL, USA), and R, version 3.6.1. *p*-values < 0.05 were considered statistically significant.

## 3. Results

### 3.1. Study Cohort

A total of 184 patients were prospectively enrolled in this study; 101 patients with IC, 23 patients with *S. aureus* bacteremia, 28 patients with *E. coli* bacteremia, and 32 healthy controls. Demographic data and baseline characteristics of all groups are depicted in Table 1.

Patients with IC presented significantly higher rates of recent abdominal surgery than patients with bacteremia or healthy controls (*p* < 0.001) Of the 101 IC patients, 41 (40.6%) had an active hemato-oncological disease, compared to 2/23 (8.7%) and 5/28 (17.9%) of patients with *S. aureus* and *E. coli* bacteremia, respectively (*p* 0.001). The majority of IC patients with malignant diseases presented with solid tumors (85.4%), whereas 60% of patients with *E. coli* bacteremia had hematological malignancies. Eighteen out of the 101 (17.8%) IC patients had immunosuppressive therapy, compared to 2/23 (8.7%) of patients with *S. aureus* bacteremia, and 6/28 (21.4%) of patients with *E. coli* bacteremia. In patients with IC, the most frequent immunosuppressive agents included corticosteroids (38.9%), calcineurin inhibitors (27.8%), and mycophenolate mofetil (22.2%).

Table 2 presents detailed characteristics of the 101 patients with IC. Eighty-six (85.1%) patients with IC were enrolled in center 1, 8 (7.9%) in center 2, and 7 (6.9%) in center 3. A total of 62 (61.4%) cases were classified as proven IC, 34 (33.7%) as probable IC, and 5 (5%) as possible IC. Candidemia was detected in 71 (70.3%) IC patients; of these 56 (78.9%) were identified as true candidemia cases. Isolated *Candida* species of patients with IC included *C. albicans* (69.3%), *C. glabrata* (12.9%), *C. parapsilosis* (7.9%), >1 *Candida* spp. (5.9%), and other *Candida* spp. (4%). Follow-up blood cultures were performed in 40/56 (71.4%) patients with candidemia (total number) and 27/41 (65.9%) patients with true candidemia, with a positivity rate of 17.4% (7/40) and 25.9% (7/27), respectively (data only available for center 1, Graz).

Clinical characteristics of IC and bacteremic patients are presented in Table 3. Both the duration of hospitalization prior to index sampling and the duration of ICU stay prior to index sampling were significantly longer in patients with IC compared to patients with bacteremia (*p* < 0.001). No significant differences were observed regarding levels of inflammation markers (CRP, PCT) on the day of index sampling. BDG testing was performed at the discretion of the attending physician and was available in 55/86 (64.0%) patients with IC from center 1. The highest BDG positivity rate (i.e., number of positive BDG values (≥80 pg/mL) related to number of BDG measurements) was observed on the day of index sampling, although 4/13 patients with IC presented positive BDG values as early as one week prior to index sampling. Vasopressor therapy, sedation, treatment with morphine, and parenteral nutrition were significantly more frequent in patients with invasive *Candida* infections compared to patients with bacteremia (*p* < 0.001).Intravascular catheters (e.g., central venous catheters, femoral vascular access), nasogastric tube, urinary catheter, and respiratory support (invasive mechanical ventilation), as well as non-invasive ventilation (NIV)) prior to index sampling, were also significantly more common in patients with IC (*p* < 0.001). The median duration of antibiotic therapy prior to index sampling was 12 (range 0–145) days for patients with IC compared to 0 days in bacteremic patients (*p* < 0.001). In patients with *E. coli* bacteremia, antibiotic therapy was initiated at the sampling date of index cultures in 22/28 (79%) patients, at later time points in 3/28 (11%) patients and was preexisting in 3/28 (11%). Similarly, in patients with *S. aureus* bacteremia antibiotic therapy was initiated at the day of index sampling in 17/23 (74%) patients, at later time points in 3/23 (13%) patients and was preexisting in 3/23 (13%).

Hospital mortality rate was 39.1% for patients with *S. aureus* bacteremia, compared to 28.7% for patients with IC, and 3.6% for patients with *E. coli* bacteremia. In 55.2% of deceased IC patients death was attributable to IC.

### 3.2. Cytokine Measurements

#### 3.2.1. IL-17A

**Figure 1:**
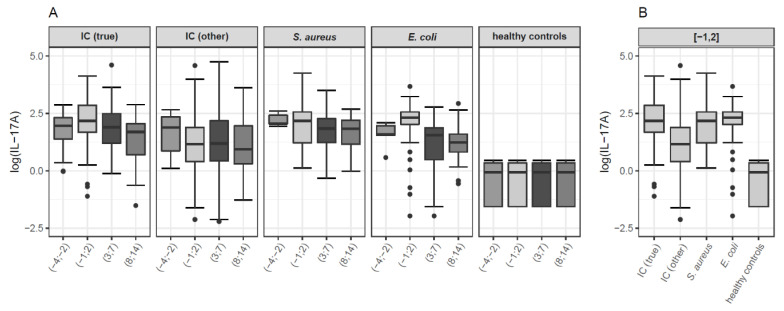
Boxplots of interleukin 17A (IL-17A) values of the investigated study groups for each time interval (**A**), and with focus on time interval (−1; 2) (**B**) for the total study population. IL-17A values (pg/mL) are depicted on the logarithmic scale. In the boxplots, median values are depicted as bold line and the box spans from first to third quartile; whiskers extend to a maximum of 1.5 times the interquartile range (IQR, third minus first quartile) out from the respective box end; all remaining values are indicated as dots. Time intervals represent study days. Time interval (−1; 2) includes blood samples collected one day prior until up to two days after collection of the index sample. IC = invasive candidiasis. IC (true) = patients with true candidemia (*n* number of patients in boxplot B = 46, *n* number of samples in boxplot B = 97). IC (other) = patients with candidemia of unclear significance (positive blood cultures from central veins only), proven IC other than candidemia, and patients with probable and possible IC (*n* number of patients in boxplot B = 41, *n* number of samples in boxplot B = 81). *S. aureus* = patients with *S. aureus* bacteremia (*n* number of patients in boxplot B = 22, *n* number of samples in boxplot B = 45). *E. coli* = patients with *E. coli* bacteremia (*n* number of patients in boxplot B = 27, *n* number of samples in boxplot B = 48).

Interleukin 17A values of the five investigated study groups (IC (true), IC (other), *S. aureus* bacteremia, *E. coli* bacteremia, healthy controls) per time interval are shown in boxplots in Figure 1. Table A2 presents pairwise group comparisons at each time point for the total study population.

Descriptive statistics of IL-17A levels of the respective study groups for the respective time intervals are depicted in Table A3.

Overall, IL-17A levels were significantly higher in all patient groups (IC (true), IC (other), *S. aureus* bacteremia, *E. coli* bacteremia) compared to healthy controls for each of the defined time intervals. There was a significant difference in IL-17A values between patients with IC (true) and IC (other) for the time intervals (−1; 2) and (3; 7), with significantly higher IL-17A values in patients with true candidemia (*p* < 0.001 for both comparisons; see Table A2 for details). Concerning time intervals (−1; 2) and (3; 7), patients with *S. aureus* bacteremia showed significantly higher IL-17A levels compared to patients with IC (other) (*p* < 0.02 for both comparisons). In contrast, no significant differences in IL-17A values could be observed between patients with true candidemia and *S. aureus* bacteremia for any of the investigated time intervals. Regarding time interval (−1; 2), patients with IC (other) had significantly lower IL-17A levels than patients with *E. coli* bacteremia (*p* < 0.001). During study days (3; 7), patients with true candidemia presented significantly higher IL-17A values than patients with *E. coli* bacteremia (*p* 0.008), whereas this finding could not be observed for the other investigated time intervals (see Table A2 for details).

Exclusion of immunocompromised patients and patients with hematological malignancies (i.e., sensitivity analysis) did not affect these findings (data not shown).

Within the study group IC (true), IL-17A values were significantly lower in the earliest investigated time interval (−4; −2) compared to (−1; 2); and compared to (3; 7), respectively. No significant differences were observed for the comparisons of time intervals (−4; −2) versus (8; 14), and (−1; 2) versus (3; 7). IL-17A values decreased in later study days. Accordingly, IL-17A levels were lower in (8; 14) versus (−1; 2), and (8;14) versus (3; 7) (*p* < 0.001 for both comparisons) (Table A4).

#### 3.2.2. Other Cytokines (IL-6, IL-8, IL-10, IL-17F, IL-22, IL-23 (p19), IFN-γ, TNF-α, PTX3, TGF-β)

**Figure 2:**
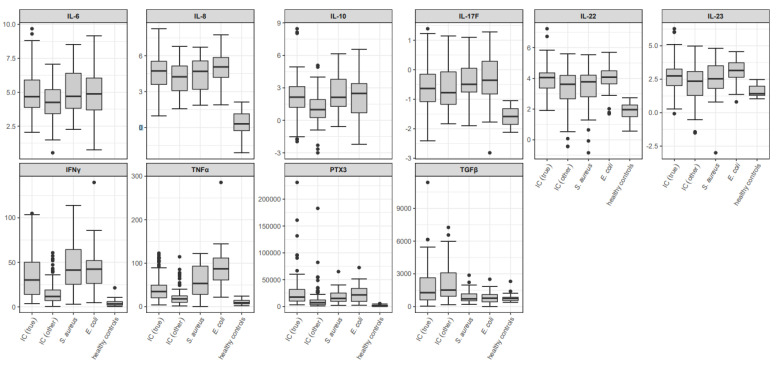
Boxplots of designated cytokine values (IL-6, IL-8, IL-10, IL-17F, IL-22, IL-23, IFN-γ, TNF-α, PTX3, TGF-β) for the total study population at time interval (−1; 2) relative to index sampling. Values of interleukin parameters are depicted on the logarithmic scale; other values are given in pg/mL. In the boxplots, median values are depicted as bold line and the box spans from first to third quartile; whiskers extend to a maximum of 1.5 times the interquartile range (IQR, third minus first quartile) out from the respective box end; all remaining values are indicated as dots. IL = interleukin. IFN-γ = interferon-γ. PTX3 = Pentraxin-related protein 3. TNF-α = tumor necrosis factor-α. TGF-β = transforming growth factor-β. IC = invasive candidiasis. IC (true) = patients with true candidemia. IC (other) = patients with candidemia of unclear significance (positive blood cultures from central veins only), proven IC other than candidemia, and patients with probable and possible IC. *S. aureus* = patients with *S. aureus* bacteremia. *E. coli* = patients with *E. coli* bacteremia.

Boxplots of other cytokine values of the different study groups at time interval (−1; 2) relative to index sampling are shown in Figure 2.

Table 4 depicts *p*-values obtained from comparisons of cytokine values other than IL-17A (IL-6, IL-8, IL-10, IL-17F, IL-22, IL-23, IFN-γ, TNF-α, PTX3, TGF-β) between the different study groups at time interval (−1; 2).

A detailed description of observed differences between investigated cytokine levels is available in the Appendix A (“Detailed description of “Table A4. Comparison of cytokine values other than interleukin 17A (IL-6, IL-8, IL-10, IL-17F, IL-22, IL-23, IFN-γ, TNF-α, PTX3, TGF-β) between the different study groups at time interval (−1; 2) (*p*-values)” (see Section 3.2.1. in the main text)”).

Overall, the comparison of IL-10, IL-22, IL-8, IFN-γ, PTX3 and TNF-α values between IC (true) and IC (other) yielded significantly higher cytokine values for patients with true candidemia, whereas no significant differences could be observed for the other investigated cytokines.

Median IL-22 levels were significantly higher in patients with true candidemia compared to patients with *S. aureus* bacteremia, while no significant differences could be detected for the comparison of all other cytokine values.

In contrast, patients with *S. aureus* bacteremia presented significantly elevated IL-10, IFN-γ, PTX3 and TNF-α levels compared to IC (other) patients.

Comparison of median cytokine levels between patients with true candidemia and patients with *E. coli* bacteremia showed significantly elevated TNF-α levels in bacteremic patients, whereas TGF-β levels were significantly elevated in IC (true) patients. On the other hand, *E. coli* patients had significantly higher IL-10, IL-22, IL-23, IL-6, IL-8, IFN-γ, PTX3 and TNF-α values than patients with IC (other).

Patients with true candidemia and patients with IC (other) presented significantly elevated TGF-β values compared to bacteremic patients, except for the comparison of IC (true) and *S. aureus*. Exclusion of immunocompromised patients and patients with hematological malignancies resulted in significant differences of TGF-β levels between IC (true) and bacteremic patients for time intervals (−4; −2), (−1; 2) and (3; 7), with significantly higher values in candidemic patients.

### 3.3. Kyurenine and Tryptophan Concentrations

**Figure 3:**
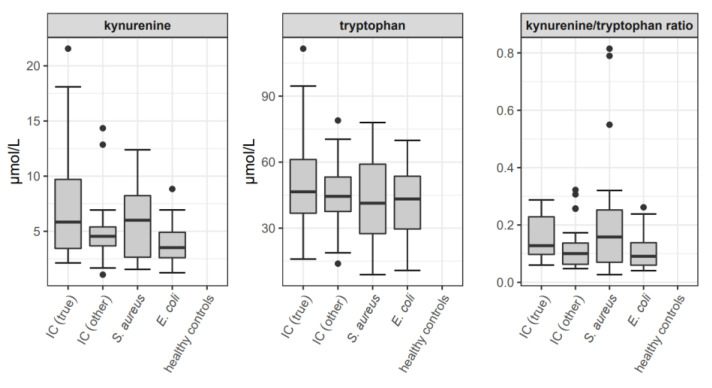
Boxplots of kynurenine and tryptophan concentrations as well as the kynurenine/tryptophan ratio for the total study population at time interval (−1;2) (relative to index sampling). Except for the ratio, values are given in µmol/L. In the boxplots, median values are depicted as bold line and the box spans from first to third quartile; whiskers extend to a maximum of 1.5 times the interquartile range (IQR, third minus first quartile) out from the respective box end; all remaining values are indicated as dots. IC = invasive candidiasis. IC (true) = patients with true candidemia. IC (other) = patients with candidemia of unclear significance (positive blood cultures from central veins only), proven IC other than candidemia, and patients with probable and possible IC. *S. aureus* = patients with *S. aureus* bacteremia. *E. coli* = patients with *E. coli* bacteremia.

Boxplots of kynurenine and tryptophan concentrations as well as of the kynurenine/tryptophan ratio of the total study population for the time interval (−1; 2) are depicted in Figure 3.

Referring to kynurenine levels, the only statistically significant difference for time interval (−1; 2) was observed between patients with IC (true) (median 5.8, range 2.1–21.6 pg/mL) and patients with *E. coli* bacteremia (median 3.5, range 1.2–8.8 pg/mL), with significantly elevated kynurenine values in true candidemic patients (*p* 0.023).

## 4. Discussion

Recently, significantly elevated IL-17A levels in candidemic patients compared to non-candidemic patients were reported, suggesting IL-17A as potential biomarker for differentiation between *Candida*-infected and non-infected patients [21]. This observation was further supported by an observational, prospective clinical trial, showing significantly elevated IL-17A levels in patients with *Candida* sepsis compared to patients with bacterial sepsis or septic patients with fungal colonization [22].

In contrast to previous studies, which investigated cytokine levels, at the earliest, from the date of clinical suspicion of sepsis [22,32,33], we aimed to measure cytokine values at earlier time points, preferably even before sampling of index cultures. With this approach, we intended to evaluate IL-17A, as well as other cytokines involved in anti-*Candida* host response, as potential biomarker for timely anticipation of IC.

Overall, our study found that IL-17A levels were significantly elevated in all patient groups (IC, *S. aureus*, *E. coli*) compared to healthy controls. Furthermore, patients with true candidemia presented significantly higher IL-17A values compared to IC (other) for study intervals (−1; 2) and (3; 7) relative to sampling of the index culture, respectively. In comparison with bacteremic patients, however, IL-17A values were not discriminative. In fact, we could not demonstrate significant differences in IL-17A values between patients with invasive *Candida* infection and *S. aureus* bacteremia. IL-17A levels were significantly lower in patients with *E. coli* bacteremia when compared to patients with true candidemia at study interval (3; 7), whereas this finding could not be observed for the other investigated study intervals.

These findings are in contrast to our previous study [21], which also included patients with invasive *Candida* and bacterial infections, yet only a small proportion of these patients was bacteremic at the time point of sampling. In fact, only 3/20 patients with extrapulmonary infection and 3/24 patients with pneumonia had concomitant bacteremia [21]. In comparison, comparator groups with bacterial infections in this present study solely included bacteremic patients.

In a previous study [32], IL-17A values were significantly higher in candidemic patients compared to patients with gram-positive blood stream infections (BSI), whereas, as observed in our study, no significant differences could be detected between candidemic patients and patients with gram-negative BSI. In contrast, another study showed significantly higher IL-17 levels in candidemic patients compared to both patients with bacterial sepsis (gram-negative and gram-positive), and polymicrobial sepsis (i.e., bacteria and *Candida* spp. positive blood cultures) [33]. The highest IL-17 values were observed in patients with polymicrobial sepsis, although sample size was rather small (*n* = 11). Similar to our study, candidemic patients had a wide range of IL-17 values (minimum 0, maximum 611.6 pg/mL) compared to a considerably smaller range for bacteremic patients (minimum 0, maximum 103.8 pg/mL) [33]. In contrast to our study, however, the study by Akin et al. [33] compared cytokine levels between candidemic and bacteremic patients from different time points. In fact, the median time of serum sample collection for consecutive cytokine measurements was three days for candidemic patients, whereas blood culture and serum sampling were concomitantly performed in bacteremic patients [33]. In comparison, in the study by Li et al. [32], blood sample collection was performed in a comparable procedure for patients with candidemia and those with bacteremia, though only samples from a 48 h time frame after hospital admission were available for all patients.

For many of the investigated cytokines, values were significantly elevated in patients with true candidemia compared to IC (other). As described above, some of the study patients with candidemia presented *Candida* positive blood cultures from central veins only. Since diagnostic methods for determination of catheter-related bloodstream infection did not prove candidemia originating from the catheter, those cases were classified as “IC (other)”. Therefore, the study group IC (other) comprised patients with candidemia of unclear significance (positive blood cultures from central veins only), proven IC other than candidemia, and patients with probable and possible IC.

With regard to other cytokines proposed to be involved in anti-*Candida* host defense, the comparison of TGF-β levels between patients with *Candida* infection and bacteremic patients yielded especially interesting results. TGF-β induces the development of Th17 cells after stimulation with IL-6 [33]. In our study, levels of TGF-β were shown to be significantly elevated in patients with IC (other) compared to bacteremic patients for time intervals (−1; 2), (3; 7) and (8; 14). In contrast, patients with true candidemia only presented significantly elevated TGF-β values when compared to patients with *E. coli* bacteremia [for time intervals (−4; −2), (−1; 2) and (3; 7)], but not *S. aureus* bacteremia. However, exclusion of immunocompromised patients and patients with hematological malignancies resulted in significant differences of TGF-β levels between IC (true) and bacteremic patients for time intervals (−4; −2), (−1; 2) and (3; 7), with significantly higher values in candidemic patients. This finding is in line with previous data showing significantly elevated TGF-β levels in candidemic patients compared to bacteremic patients and healthy controls at unspecified time points [33].

*C. albicans* was considered to dampen immune response via modulation of tryptophan-kynurenine metabolism in order to enable colonization of the host [7,21,28]. A specific downregulation of host IL-17 secretion has been demonstrated, when peripheral blood mononuclear cells (PBMCs) were co-cultured with heat-killed and live *C. albicans* [7]. The IL-17-diminishing effect of live *C. albicans* is mediated by modulatory effects on host tryptophan metabolism. Via blockade of indoleamine-2-3-dioxygenase (IDO) expression, live *C. albicans* actively shifts tryptophan metabolism away from kynurenines and toward 5-hydroxytryptophan metabolites. Increased 5-hydroxytryptophan levels in turn lead to inhibition of further IL-17 production [7,21,28]. Referring to these in vitro observations, we hypothesized elevated levels of both IL-17A and kynurenine in patients with IC. In line with this, patients with IC had significantly higher levels of IL-17A and kynurenine compared to non-candidemic patients in our recent study [21]. In our current study, however, the only statistically significant difference in kynurenine levels was observed around the time of index sampling (−1; 2) between patients with IC (true) and *E. coli* bacteremia, with significantly higher kynurenine values in candidemic patients. Since kynurenine is a metabolite of the essential amino acid tryptophan, which is obtained from diet [36], a kynurenine/tryptophan ratio was additionally calculated in order to exclude dietary influence. Comparison of kynurenine/tryptophan ratio did not detect any additional significant differences.

Previously, a strong induction of IL-23 expression and a low production of IL-12 p70 elicited by zymosan in opposite to lipopolysaccharide (LPS) was shown and may explain the IL-23 polarization and Th17 host response to *Candida* infections [37]. In our study we did not find any difference in IL-23 values between candidemic and bacteremic patients. We did not investigate IL-12 p70 values and therefore cannot support the current notion that IL-12 p70 is inhibited by beta-glucans in candidemic patients.

## 5. Conclusions

We did not observe a discriminative competence between fungal and bacterial infections for both IL-17A and kynurenine in this study. Following this, IL-17A may be valuable as a biomarker for either fungal or bacterial blood stream infection rather than solely for invasive *Candida* infection. Further, we detected significantly elevated TGF-β levels in patients with IC compared to bacteremic patients, proposing a potential significance of TGF-β for differentiation between bacterial and *Candida* infections. However, larger studies are warranted to investigate this association.

## Figures and Tables

**Table 1:** Demographic data and baseline characteristics.

Variable	Invasive Candidiasis	*S. aureus* Bacteremia	*E. coli* Bacteremia	Healthy Controls	*p*
No. of Patients	101	23	28	32	
Age, Years; Median (Range)	64 (18–91)	69 (52–87)	79 (18–96)	46 (19–86)	<0.001
Sex					0.012
Male	64 (63.4)	17 (73.9)	10 (35.7)	15 (46.9)	
Female	37 (36.6)	6 (26.1)	18 (64.3)	17 (53.1)	
Cause of Hospital Admission					
Elective Surgery	24 (23.8)	2 (8.7)	0	32 (100)	
Infectious Disease	24 (23.8)	10 (43.5)	14 (50)		
Gastrointestinal Disease	20 (19.8)	1 (4.3)	7 (25)		
Malignant Disease	16 (15.8)	1 (4.3)	3 (10.7)		
Cardiovascular Disease	4 (4.0)	4 (17.4)	3 (10.7)		
Other	13 (12.9)	5 (21.7)	1 (3.6)		
Ward					
Internal Medicine	13 (12.9)	13 (56.5)	25 (89.3)		
Surgery	27 (26.7)	4 (17.4)	1 (3.6)	32 (100)	
Icu	44 (43.6)	4 (17.4)	1 (3.6)		
Other or Unknown	17 (16.8)	2 (8.7)	1 (3.6)		
Recent Abdominal Surgery	50 (49.5)	1 (4.3)	0	0	<0.001
Recent Other Surgery	42 (41.6)	5 (21.7)	5 (17.9)	0	
Active Hemato-oncological Disease	41 (40.6)	2 (8.7)	5 (17.9)	0	0.001
Solid Tumor	35/41 (85.4)	1/2 (50)	2/5 (40)		
Hematological Malignancy	6/41 (14.6)	1/2 (50)	3/5 (60)		
Immunosuppressive Therapy	18 (17.8)	2 (8.7)	6 (21.4)	0	

Data are no. (%) of patients, unless otherwise indicated. ICU = intensive care unit.

**Table 2:** Characteristics of patients with invasive candidiasis.

Variable	No. (%) of Patients
Overall Study Cohort	101 (100)
Center 1 (Graz)	86 (85.1)
Center 2 (Innsbruck)	8 (7.9)
Center 3 (Cologne)	7 (6.9)
Category	
Proven IC	62 (61.4)
Probable IC	34 (33.7)
Possible IC	5 (5.0)
Candidemia	
Total Number	71 (70.3)
True Candidemia ^1^	56 (55.4)
*Candida* Species	
*C. albicans*	70 (69.3)
*C. glabrata*	13 (12.9)
*C. parapsilosis*	8 (7.9)
>1 *Candida* spp.	6 (5.9)
Other *Candida* spp.	4 (4.0)
Follow-Up Blood Cultures ^2^	
Candidemia (Total Number) ^3^	40/56 (71.4)
True Candidemia ^4^	27/41 (65.9)
Positivity Rate of Follow-Up Blood Cultures ^2^	
Candidemia (Total Number) ^5^	7/40 (17.5)
True Candidemia ^6^	7/27 (25.9)

IC = invasive candidiasis; ^1^ positive peripheral blood cultures +/− positive central blood cultures (exclusion of positive blood cultures from central veins only); ^2^ Data only available for center 1 (Graz); ^3^ number of patients with *Candida* follow-up blood cultures performed relative to total number of candidemic patients; ^4^ number of patients with *Candida* follow-up blood cultures performed relative to number of patients with true candidemia; ^5^ number of patients with ≥1 positive follow-up blood culture(s) relative to total number of candidemic patients; ^6^ number of patients with ≥1 positive follow-up blood culture(s) relative to number of patients with true candidemia.

**Table 3:** Clinical characteristics of patients with invasive candidiasis, *S. aureus* bacteremia and *E. coli* bacteremia.

Variable	Invasive Candidiasis	*S. aureus* Bacteremia	*E. coli* Bacteremia	*p*
No. of Patients	101	23	28	
Duration of In-Hospital Stay Prior to Index Sampling, Days	16 (0–98)	1 (0–24)	0 (0–52)	**<0.001**
Duration of ICU Stay Prior to Index Sampling, Days	5 (0–121)	0 (0–5)	0 (0–0)	**<0.001**
Laboratory Values on Day of Index Sampling				
CRP (Mg/L)	132 (3–521)	146 (5–472)	93 (1–399)	0.226
PCT (Ng/mL)	1.0 (0.1–109.8)	0.95 (0.2–358.8)	5.2 (0.2–84.9)	0.351
Positive BDG (≥80 pg/mL) *	31/86 (36.0)	n.a.	n.a.	
Within 1 Week Prior to Index Sampling ^1^	4/13 (30.8)	n.a.	n.a.	
On Index Sampling Day ^1^	9/15 (60.0)	n.a.	n.a.	
Within 1 Week after Index Sampling ^1^	21/43 (48.8)	n.a.	n.a.	
Vasopressors ^2,^*	28/86 (32.6)	1 (4.3)	1 (3.6)	**<0.001**
Proton-Pump Inhibitors *	65/86 (75.6)	13 (56.5)	15 (53.6)	**0.024**
Sedation ^3,^*	28/86 (32.6)	4 (17.4)	0	**<0.001**
Morphine ^*^	45/86 (52.3)	5 (21.7)	6 (21.4)	**<0.001**
Parenteral Nutrition *	38/86 (44.2)	0	0	**<0.001**
Duration of Parenteral Nutrition Prior to Index Sampling, Days	8 (0–65)	-	-	
Nasogastric Tube *	39/86 (45.3)	1 (4.3)	0	**<0.001**
Number of Central Venous Catheters *	1 (0–3)	0 (0–2)	0 (0–1)	**<0.001**
Femoral Vascular Access *	12/86 (14)	0	0	**0.016**
Urinary Catheter *	59/86 (68.6)	7 (30.4)	6 (21.4)	**<0.001**
Duration of Fever Prior to *Candida* spp./*S. aureus*/*E. coli* Detection, Days *	0 (0–16)	1 (0–3)	1 (0–7)	0.095
Oxygenation Index *	253 (139–523)	280	n.a.	
Respiratory Support *				
Invasive Mechanical Ventilation Prior to Index Sampling	38/86 (44.2)	1 (4.3)	0	**<0.001**
Duration of Mechanical Ventilation Prior to Index Sampling, Days	7 (1–49)	1 (1)	-	
NIV Prior to Index Sampling	23/86 (26.7)	0	0	**<0.001**
Duration of NIV Prior to Index Sampling	3 (1–15)	-	-	
Duration of Mechanical Ventilation Starting From Index Sampling Until end of Ventilation, Days	0 (0–72)	0 (0–28)	0 (0–2)	**0.018**
Severity of Illness				
SAPS3 Score ^4^	54 (19–98)	62 (31–72)	44 (34–53)	0.616
SAPS2 Score ^5^	29 (21–53)	-	-	
SOFA Score Minimum ^6,^*	2 (0–11)	2 (0–8)	2 (0–6)	0.769
Antibiotic Therapy				
Duration of Antibiotic Therapy Prior To Index Sampling, Days	12 (0–145)	0 (0–5)	0 (0–51)	**<0.001**
Antibiotic Therapy During Index Sampling	85 (84.2)	6 (26.1)	3 (10.7)	**<0.001**
Antibiotic Therapy After Index Sampling	96 (95)	23 (100)	28 (100)	1.0
Antifungal Therapy				
Antifungal Therapy Within 8 Weeks Prior To Inclusion	15 (14.9)	0	0	
Antifungal Therapy During Index Sampling	11 (10.9)	0	2 (7.1)	0.308
Antifungal Therapy after Index Sampling	91 (90.1)	0	0	
Bacteremia	10 (9.9)	23 (100)	28 (100)	**<0.001**
Other Invasive Bacterial Infection *	36/86 (41.9)	2 (8.7)	4 (14.3)	**0.001**
Viremia ^7^	9/33 (27.3)	0/2	1/7 (14.3)	0.327
Outcome				
Intrahospital Death	29/101 (28.7)	9 (39.1)	1 (3.6)	**0.008**
Death Related to IC	16/29 (55.2)	-	-	
Death Possibly Related to IC	1/29 (3.4)	-	-	
Death Within 30 Days after Index Culture	24/101 (23.8)	7 (30.4)	1 (3.6)	**0.026**

Data are given as median (range) or no. (%). Bold *p*-values indicate statistically significant differences with a *p*-value less than 0.05. Characteristics were determined on day of index sampling, unless otherwise indicated. ICU = intensive care unit. CRP = C-reactive protein. PCT = procalcitonin. NIV = non-invasive ventilation. n.a. = not available/not performed. IC = invasive candidiasis. BDG = 1,3-beta-D Glucan; * Data only available for center 1 (Graz); ^1^ number of positive BDG values (≥80 pg/mL) relative to number of BDG measurements; ^2^ norepinephrine, vasopressin; ^3^ propofol, benzodiazepine; ^4^ not available for candidemic patients from center 3 (Cologne); ^5^ only for candidemic patients from center 3 (Cologne); ^6^ Due to missing data, the complete SOFA Score could not be determined for most patients, therefore a partial SOFA Score was calculated including all variables available. “SOFA Score minimum” not available for candidemic patients from center 2 (Innsbruck) and 3 (Cologne); ^7^ predominantly Herpes simplex virus and Cytomegalovirus viremia; performance of Virus-PCRs at the discretion of the attending physician.

**Table 4:** Comparison of cytokine values other than interleukin 17A (IL-6, IL-8, IL-10, IL-17F, IL-22, IL-23, IFN-γ, TNF-α, PTX3, TGF-β) between the different study groups at time interval (−1; 2) (*p*-values).

Contrast	IL-6	IL-8	IL-10	IL-17F	IL-22	IL-23	IFN-γ	TNF-α	PTX3	TGF-β
IC (true) vs. IC (other)	0.134	**0.048** **IC (true) >**	**0.025** **IC (true) >**	0.991	**0.019** **IC (true) >**	0.064	**<0.001** **IC (true) >**	**<0.001** **IC (true) >**	**<0.001** **IC (true) >**	0.07
IC (true) vs. *S. aureus*	0.52	0.509	0.359	0.34	**0.013** **IC (true) >**	0.47	0.107	0.209	0.561	0.079
IC (true) vs. *E. coli*	0.354	0.241	0.862	0.698	0.857	0.09	0.06	**<0.001** ***E. coli*** **>**	0.436	**0.01** **IC (true) >**
IC (true) vs. healthy	n.d.	**<0.001** **IC (true) >**	n.d.	0.109	**<0.001** **IC (true) >**	0.053	**<0.001** **IC (true) >**	**<0.001** **IC (true) >**	**<0.001** **IC (true) >**	**0.027** **IC (true) >**
IC (other) vs. *S. aureus*	0.069	0.335	**0.008** ***S. aureus*** **>**	0.413	0.648	0.379	**<0.001** ***S. aureus*** **>**	**<0.001** ***S. aureus*** **>**	**0.012** ***S. aureus*** **>**	**0.002** **IC (other) >**
IC (other) vs. *E. coli*	**0.03** ***E. coli*** **>**	**0.005** ***E. coli*** **>**	**0.03** ***E. coli*** **>**	0.748	**0.022** ***E. coli*** **>**	**0.001** ***E. coli*** **>**	**<0.001** ***E. coli*** **>**	**<0.001** ***E. coli*** **>**	**<0.001** ***E. coli*** **>**	**<0.001** **IC (other) >**
IC (other) vs. healthy	n.d.	**<0.001** **IC (other) >**	n.d.	0.123	**0.002** **IC (other) >**	0.33	**<0.001** **IC (other) >**	**0.001** **IC (other) >**	**<0.001** **IC (other) >**	**<0.001** **IC (other) >**
*S. aureus* vs. *E. coli*	0.843	0.114	0.479	0.556	**0.014** ***E. coli*** **>**	**0.035** ***E. coli*** **>**	0.899	**0.003** ***E. coli*** **>**	0.239	0.641
*S. aureus* vs. healthy	n.d.	**<0.001** ***S. aureus*** **>**	n.d.	0.054	**0.006** ***S. aureus*** **>**	0.149	**<0.001** ***S. aureus*** **>**	**<0.001** ***S. aureus*** **>**	**<0.001** ***S. aureus*** **>**	0.869
*E. coli* vs. healthy	n.d.	**<0.001** ***E. coli*** **>**	n.d.	0.086	**<0.001** ***E. coli*** **>**	**0.006 *E. coli* >**	**<0.001** ***E. coli*** **>**	**<0.001** ***E. coli*** **>**	**<0.001** ***E. coli*** **>**	0.737

Statistical data analysis was performed for the total study population. Bold *p*-values indicate statistically significant differences with a *p*-value less than 0.05. IC = invasive candidiasis. IC (true) = patients with true candidemia. IC (other) = patients with candidemia of unclear significance (positive blood cultures from central veins only), proven IC other than candidemia, and patients with probable and possible IC. *S. aureus* = patients with *S. aureus* bacteremia. *E. coli* = patients with *E. coli* bacteremia. healthy = healthy controls. IL = interleukin. IFN-γ = interferon-γ. PTX3 = Pentraxin-related protein 3. TNF-α = tumor necrosis factor-α. TGF-β = transforming growth factor-β. n.d. = no data.

## Data Availability

The data presented in this study are available on request from the corresponding author.

## Appendix A

**Table A1:** Diagnostic criteria for invasive candidiasis used in this study.

Category	Host Factor	Clinical Presentation	Mycological Evidence
Proven IC ^1^	not required	*Candida* species isolated from blood cultures or from other specimen obtained by a sterile procedure from a normally sterile site (including a freshly placed (<24 h ago) drain) showing a clinical or radiological abnormality consistent with an infectious disease process	
Probable IC	At least one of the following: (1) impaired gut wall integrity ^2^ (2) impaired cutaneous barriers to bloodstream infection ^3^ (3) colonization with *Candida* species in ≥2 non-sterile sites ^4^ (4) recent history of neutropenia (<500 neutrophils/mm^3^ for >10 days) temporally related to the onset of invasive fungal disease (5) hematologic malignancy (6) receipt of an allogeneic stem cell transplant or solid organ transplant (7) prolonged use of corticosteroids at a therapeutic dose of ≥0.3 mg/kg for ≥3 weeks in the past 60 days (8) treatment with other recognized T-cell immunosuppressants (e.g., calcineurin inhibitors, tumor necrosis factor-α blockers, lymphocyte-specific monoclonal antibodies, immunosuppressive nucleoside analogues) during the past 90 days (9) inherited severe immunodeficiency (e.g., chronic granulomatous disease, STAT 3 deficiency, CARD9 deficiency, STAT-1 gain of function, or severe combined immunodeficiency) (10) acute graft-versus-host disease grade III or IV involving the gut, lungs, or liver that is refractory to first-line treatment with steroids	clinical or radiological (non-pulmonary) abnormalities consistent with an infectious disease process that are otherwise unexplained	At least one of the following: (1) ß-D-glucan ≥ 80 pg/mL (Fungitell^®^) detected in at least 2 consecutive serum samples provided that other etiologies have been excluded (2) recovery of *Candida* in an adequate intra-abdominal specimen (obtained surgically or within 24 h from external drainage)

^1^ Since histopathological examinations require a certain time period, histopathological evidence of IC is usually retrospective in nature, making timely acquisition of blood samples unfeasible. Following that, histopathological evidence of IC was not included as diagnostic criterion of proven IC in our study. ^2^ recent abdominal surgery, biliary tree abnormality, recurrent intestinal perforations, ascites, mucositis, severe pancreatitis, parenteral nutrition. ^3^ presence of central vascular access device, hemodialysis. ^4^ respiratory tract secretions, stool, skin, wound sites, urine, drains that have been in place for ≥24 h. Reproduced from [23] and from previously unpublished work of Bassetti et al., TIMM 2015.

**Table A2:** Differences in interleukin 17A levels between study groups per time interval for the total study population.

Time Interval (Study Days)	Contrast	Estimate (Lower, Upper CI)	*p*
(−4;−2)	IC (true)—IC (other)	0.39 (−0.22, 1.00)	0.205
	IC (true)—*S. aureus*	−0.43 (−1.55, 0.68)	0.436
	IC (true)—*E. coli*	0.38 (−0.46, 1.22)	0.365
	IC (true)—healthy	2.30 (1.53, 3.07)	**<0.001**
	IC (other)—*S. aureus*	−0.82 (−2.01, 0.37)	0.169
	IC (other)—*E. coli*	−0.01 (−0.94, 0.92)	0.986
	IC (other)—healthy	1.91 (1.05, 2.78)	**<0.001**
	*S. aureus—E. coli*	0.81 (−0.51, 2.13)	0.219
	*S. aureus*—healthy	2.73 (1.46, 4.01)	**<0.001**
	*E. coli*—healthy	1.92 (0.88, 2.96)	**0.001**
(−1;2)	IC (true)—IC (other)	0.93 (0.49, 1.38)	**<0.001**
	IC (true)—*S. aureus*	0.11 (−0.43, 0.64)	0.694
	IC (true)—*E. coli*	−0.07 (−0.57, 0.43)	0.785
	IC (true)—healthy	2.49 (1.46, 3.51)	**<0.001**
	IC (other)—*S. aureus*	−0.83 (−1.37, −0.28)	**0.003**
	IC (other)—*E. coli*	−1.00 (−1.52, −0.49)	**<0.001**
	IC (other)—healthy	1.55 (0.52, 2.58)	**0.003**
	*S. aureus—E. coli*	−0.18 (−0.77, 0.42)	0.559
	*S. aureus*—healthy	2.38 (1.31, 3.45)	**<0.001**
	*E. coli*—healthy	2.56 (1.50, 3.61)	**<0.001**
(3;7)	IC (true)—IC (other)	0.99 (0.52, 1.46)	**<0.001**
	IC (true)—*S. aureus*	0.29 (−0.28, 0.86)	0.317
	IC (true)—*E. coli*	0.75 (0.20, 1.30)	**0.008**
	IC (true)—healthy	2.38 (1.34, 3.43)	**<0.001**
	IC (other)—*S. aureus*	−0.70 (−1.28, −0.12)	**0.019**
	IC (other)—*E. coli*	−0.24 (−0.80, 0.32)	0.405
	IC (other)—healthy	1.39 (0.34, 2.44)	**0.010**
	*S. aureus—E. coli*	0.46 (−0.18, 1.11)	0.159
	*S. aureus*—healthy	2.09 (0.99, 3.19)	**<0.001**
	*E. coli*—healthy	1.63 (0.54, 2.72)	**0.004**
(8;14)	IC (true)—IC (other)	0.33 (−0.27, 0.94)	0.276
	IC (true)—*S. aureus*	−0.25 (−0.87, 0.37)	0.425
	IC (true)—*E. coli*	−0.07 (−0.68, 0.54)	0.821
	IC (true)—healthy	1.72 (0.76, 2.69)	**0.001**
	IC (other)—*S. aureus*	−0.58 (−1.19, 0.03)	0.061
	IC (other)—*E. coli*	−0.40 (−1.01, 0.20)	0.187
	IC (other)—healthy	1.39 (0.43, 2.35)	**0.005**
	*S. aureus—E. coli*	0.18 (−0.44, 0.80)	0.563
	*S. aureus*—healthy	1.97 (1.01, 2.94)	**<0.001**
	*E. coli*—healthy	1.79 (0.83, 2.75)	**<0.001**

Estimates are depicted on the logarithmic scale. Bold *p*-values indicate statistically significant differences with a *p*-value less than 0.05. CI = confidence interval. IC = invasive candidiasis. IC (true) = patients with true candidemia. IC (other) = patients with candidemia of unclear significance (positive blood cultures from central veins only), proven IC other than candidemia, and patients with probable and possible IC. *S. aureus* = patients with *S. aureus* bacteremia. *E. coli* = patients with *E. coli* bacteremia. healthy = healthy controls.

**Table A3:** Descriptive statistics of interleukin 17A levels.

Time Interval	Study Group	Mean	SD	Median	IQR
(−4;−2)	IC (true)	7.6	5.1	7.1	4.0–10.0
	IC (other)	6.7	4.6	6.6	2.4–10.6
	*S. aureus*	9.4	2.8	7.7	7.7–11.2
	*E. coli*	5.3	2.5	4.8	4.7–7.1
	healthy controls	0.9	0.6	0.9	0.2–1.4
(−1;2)	IC (true)	11.9	9.9	8.8	5.4–17.3
	IC (other)	6.7	13.2	3.2	1.5–6.6
	*S. aureus*	11.6	13.1	8.8	3.4–13.0
	*E. coli*	10.8	7.1	10.1	7.5–13.0
	healthy controls	0.9	0.6	0.9	0.2–1.4
(3;7)	IC (true)	10.3	12.2	6.6	3.3–12.0
	IC (other)	9.5	17.4	3.3	1.5–8.8
	*S. aureus*	6.8	4.9	6.3	3.4–9.7
	*E. coli*	4.7	3.4	4.7	1.6–6.5
	healthy controls	0.9	0.6	0.9	0.2–1.4
(8;14)	IC (true)	5.6	4.2	5.4	2.0–7.8
	IC (other)	7.1	9.7	2.5	1.4–7.1
	*S. aureus*	6.3	3.3	6.2	3.2–9.1
	*E. coli*	4.6	4.1	3.4	2.3–4.9
	healthy controls	0.9	0.6	0.9	0.2–1.4

Values are given in pg/mL. Time intervals represent study days. SD = standard deviation. IQR = interquartile range. IC (true) = patients with true candidemia. IC (other) = patients with candidemia of unclear significance (positive blood cultures from central veins only), proven IC other than candidemia, and patients with probable and possible IC. *S. aureus* = patients with *S. aureus* bacteremia. *E. coli* = patients with *E. coli* bacteremia.

**Table A4:** Comparison of cytokine values other than interleukin 17A (IL-6, IL-8, IL-10, IL-17F, IL-22, IL-23, IFN-γ, TNF-α, PTX3, TGF-β) between the different study groups at time interval (−1;2) (*p*-values) (see Section 3.2.1).

Comparison of Time Intervals	Estimate (Lower, Upper CI)	*p*
(−4;−2) vs. (−1;2)	−0.50 (−0.74, −0.26)	**<0.001**
(−4;−2) vs. (3;7)	−0.45 (−0.71, −0.20)	**0.001**
(−4;−2) vs. (8;14)	−0.01 (−0.30, 0.28)	0.961
(−1;2) vs. (3;7)	0.05 (−0.11, 0.21)	0.553
(−1;2) vs. (8;14)	0.49 (0.28, 0.70)	**<0.001**
(3;7) vs. (8;14)	0.44 (0.25, 0.63)	**<0.001**

Estimates are depicted in logarithmic scale. Time intervals represent study days. IC = invasive candidiasis. IC (true) = patients with true candidemia. CI = confidence interval.

Concerning IL-6, the only statistically significant difference for time interval (−1;2) was observed between patients with *E. coli* bacteremia (median 131.6, IQR 40.3–428.3 pg/mL) and IC (other) (median 70.4, IQR 30.7–179.7 pg/mL), with significantly higher IL-6 values in bacteremic patients (*p* 0.03).

Median IL-8 values were significantly higher in all patient groups compared to healthy controls (*p* < 0.05 for all comparisons). Patients with IC (true) presented significantly higher IL-8 levels (median 113.2, IQR 36.4–253.7 pg/mL) than patients with IC (other) (median 69.3, IQR 21.9–171.9 pg/mL, *p* 0.048). Further, median IL-8 values were significantly elevated in patients with *E. coli* bacteremia (median 159.2, IQR 66.2–348.3 pg/mL) compared to IC (other) (*p* 0.005). No significant differences could be detected between patients with true candidemia and bacteremic patients.

Median IL-10 levels were significantly elevated in patients with IC (true) (median 8.5, IQR 3.3–22.6 pg/mL) compared to IC (other) (median 2.7, IQR 1.3–6.8 pg/mL *p* 0.025). No significant differences could be observed between patients with true candidemia and bacteremic patients, whereas IL-10 levels were significantly lower in patients with IC (other) compared to patients with *S. aureus* or *E. coli* bacteremia (*p* 0.008 and *p* 0.03, respectively).

Comparison of median IL-17F values yielded no significant differences between the investigated study groups for the designated time interval.

Median IL-22 levels were significantly higher in all patient groups (IC (true), IC (other), *S. aureus*, *E. coli*) compared to healthy controls (*p* < 0.05 for all comparisons). Additionally, patients with IC (true) had higher IL-22 values than patients with IC (other) and *S. aureus* bacteremia, respectively (*p* 0.019 and *p* 0.013). Patients with *E. coli* bacteremia presented higher median IL-22 levels (median 59, IQR 38.3–89.4 pg/mL) than patients with IC (other) (median 37.3, IQR 14.5–66.3 pg/mL, *p* 0.022) and *S. aureus* bacteremia (median 43.6, IQR 16.4–67.2 pg/mL, *p* 0.014).

IL-23 values were significantly elevated in patients with *E. coli* bacteremia (median 23.5, IQR 14.1–41.7 pg/mL) compared to IC (other) (median 10.5, IQR 3.6–21.8 pg/mL, *p* 0.001), *S. aureus* bacteremia (median 12.6, IQR 6.1–33.4 pg/mL, *p* 0.035), and healthy controls (median 4.2, IQR 3.6–7.5 pg/mL, *p* 0.006). No significant differences could be observed between patients with IC (true) and bacteremic patients.

Median IFN-γ levels were significantly higher in all patient groups compared to healthy controls (*p* < 0.05 for all comparisons). Additionally, patients with true candidemia showed significantly higher IFN-γ levels (median 30.2, IQR 14.0–50.1 pg/mL) than patients with IC (other) (median 11.7, IQR 7.1–19.0 pg/mL, *p* < 0.001), whereas no significant differences could be observed when compared to bacteremic patients. Patients with *S. aureus* and *E. coli* bacteremia had significantly higher IFN-γ values (*S. aureus*, median 41.3, IQR 25.4–64.4 pg/mL; *E. coli*, median 42.4, IQR 26.5–51.9 pg/mL) than patients with IC (other) (*p* < 0.001 for both comparisons).

Median TNF-α levels were significantly elevated in all patient groups compared to healthy controls (*p* < 0.05 for all comparisons). Patients with true candidemia had significantly higher TNF-α levels (median 34.9, IQR 20.6–49.5 pg/mL) than patients with IC (other) (median 18.1, IQR 10.1–25.0 pg/mL); while values were significantly lower when compared to patients with *E. coli* bacteremia (median 87.2, IQR 61.5–112.0 pg/mL, *p* < 0.001 for both comparisons). Significant differences were also observed regarding TNF-α levels of patients with *S. aureus* bacteremia (median 53.5, IQR 28.6–93.2 pg/mL) or *E. coli* bacteremia compared to IC (other), with significantly higher TNF-α levels in bacteremic patients (*p* < 0.001 for both comparisons).

As already described for other cytokines, median PTX3 values were significantly higher in all patient groups compared to healthy controls (*p* < 0.05 for all comparisons). Further, patients with IC (true) had significantly higher PTX3 levels (median 17637.7, IQR 10148.2–31830.4 pg/mL) than patients with IC (other) (median 7226.7, IQR 3658.1–12286.3 pg/mL, *p* < 0.001). No statistically significant differences could be detected between IC (true) and patients with *S. aureus* and *E. coli* bacteremia, whereas median PTX3 values were significantly elevated in bacteremic patients (*S. aureus*, median 15122.4, IQR 9655.6–25168.0 pg/mL; *E. coli*, median 21672.7, IQR 9574.0–33777.2 pg/mL) compared to IC (other) (median 7226.7, IQR 3658.1–12286.3 pg/mL).

Patients with invasive candidiasis, both IC (true) (median 1273.3, IQR 610.4–2640.8 pg/mL) and IC (other) (median 1513.1, IQR 942.8–3090.3 pg/mL), presented significantly higher TGF-β values than healthy controls (median 745.3, IQR 549.2–853.5 pg/mL, *p* < 0.05 for both comparisons). Median TGF-β levels were significantly elevated in patients with IC (other) compared to bacteremic patients (*S. aureus*, median 707.4, IQR 534.1–1165.1 pg/mL; *E. coli*, median 766.3, IQR 432.6–8.4 pg/mL), whereas patients with IC (true) only showed significantly elevated TGF-β values when compared to patients with *E. coli* bacteremia, not *S. aureus* bacteremia.
